# Bio-degradable highly fluorescent conjugated polymer nanoparticles for bio-medical imaging applications

**DOI:** 10.1038/s41467-017-00545-0

**Published:** 2017-09-07

**Authors:** Tatjana Repenko, Anne Rix, Simon Ludwanowski, Dennis Go, Fabian Kiessling, Wiltrud Lederle, Alexander J. C. Kuehne

**Affiliations:** 1 DWI-Leibniz Institute for Interactive Materials, RWTH Aachen University, Forckenbeckstraße 50, 52076 Aachen, Germany; 2 Institute for Experimental Molecular Imaging, University Clinic and Helmholtz Institute for Biomedical Engineering, RWTH Aachen University, Pauwelsstraße 30, 52074 Aachen, Germany

## Abstract

Conjugated polymer nanoparticles exhibit strong fluorescence and have been applied for biological fluorescence imaging in cell culture and in small animals. However, conjugated polymer particles are hydrophobic and often chemically inert materials with diameters ranging from below 50 nm to several microns. As such, conjugated polymer nanoparticles cannot be excreted through the renal system. This drawback has prevented their application for clinical bio-medical imaging. Here, we present fully conjugated polymer nanoparticles based on imidazole units. These nanoparticles can be bio-degraded by activated macrophages. Reactive oxygen species induce scission of the conjugated polymer backbone at the imidazole unit, leading to complete decomposition of the particles into soluble low molecular weight fragments. Furthermore, the nanoparticles can be surface functionalized for directed targeting. The approach opens a wide range of opportunities for conjugated polymer particles in the fields of medical imaging, drug-delivery, and theranostics.

## Abstract (web summary)

Conjugated polymer nanoparticles have been applied for biological fluorescence imaging in cell culture and in small animals, but cannot readily be excreted through the renal system. Here the authors show fully conjugated polymer nanoparticles based on imidazole units that can be bio-degraded by activated macrophages.

## Introduction

Conjugated polymer nanoparticles (CPNs) are compelling probes with prospective use in bio-medical imaging^1–4^. The particles can be applied for staining cells^5^ and tissue^6^ for fluorescence-based imaging techniques such as microscopy and tomography^7^. CPNs are hydrophobic, therefore, quickly taken up by cells and they exhibit low cytotoxicity^8^. Due to their π-conjugated electron system, these organic semiconductor particles are strongly fluorescent and the emission color can be tuned across the entire visible spectrum into the near infrared (NIR), simply by adjusting the co-monomer composition^9^. Furthermore, CPNs also show excellent performance as photoacoustic contrast agents and for image-guided photodynamic and photothermal therapy^7, 10, 11^. Because the entire CPN consists of semiconducting material, their fluorescence is extremely photostable, allowing detection over long observation times^5, 12^. Furthermore, CPNs can be surface functionalized with biological recognition entities such as peptides^12^, glycans^13^, or antibodies^14^. Such surface functionalized nanoparticles are applied to specifically target bio-molecular motifs expressed on the membrane of cells in pathological tissue for example in cancerous tumors^15, 16^. CPNs can be produced by a variety of methods ranging from dispersion polymerization protocols^12, 17–21^ to post-polymerization mini-emulsification^22, 23^ or nanoprecipitation techniques^5, 24, 25^. While direct polymerization methods deliver particles in the range of 200 to 2 µm, post-polymerization techniques can also produce much smaller nanoparticles (often termed dots) with diameters below 50 nm. However, with respect to in vivo and especially clinical applications the critical diameter for nanoparticles allowing for renal clearance is around 5-6 nm^26^. Particles in the blood stream above this size-limit will be ingested by macrophages eventually leading to accumulation in the spleen or liver^27^. If the particles cannot be degraded by macrophages, they will accumulate and persist in organs and might cause unforeseeable adverse effects^28^. To circumvent this problem, colloidal entities such as liposomes, micelles, or degradable polymers are commonly applied in vivo, which can decompose into their small molecular building blocks^29^. Several polymer particles have been developed with a variety of degradable units, which allow for chain scission^30^. The triggers for chain scission range from acidic environments where esters and hydrazones are hydrolyzed to incorporated peptide sequences, which can be cleaved enzymatically^31^. The intact entity is much larger in dimension than the pore-diameter of the kidney membranes and, therefore, provides in principle long circulation times in the body. The degraded products have considerably lower molecular weights and can easily be cleared from the body through the renal membranes. However, such colloidal entities usually suffer from a lack in satisfactory fluorescence, sufficient imaging properties, and effectual contrast. None of the classical degradable polymers provide π-conjugation and the resulting polymer particles are dielectric in nature. On the contrary, CPNs are generally non-degradable due to their inert and carbon-based π-conjugated architecture. The absence of degradability in conjugated polymer particle systems prevents their safe in vivo application. This deficiency impedes their translation into the clinic and the utilization of a class of highly potent fluorescent and photo-acoustically active materials, which could enhance contrast, obviate photo-bleaching and augment signal-to-noise ratios for biological and medical imaging technologies^1, 3^. To achieve bio-degradability in fluorescent CPNs, we have to apply a conjugated moiety, which can be cleaved. However, such units are currently not known and unavailable for conjugated polymers. When looking at nature, biological light emitting units often contain an imidazole ring. Examples for these molecules are coelenterazine, vargulin^32, 33^, and the green fluorescent protein GFP^34^. The light emitting unit is generated by auto-oxidation resulting in fusion of aminoacids (such as π-conjugated phenylalanine, tyrosine with non-conjugated serine, and glycine) producing the imidazole unit. The emitting units are biodegradable through further oxidation and scission of the imidazole ring, affording again aminoacids as well as amides, which can be further metabolized^35–37^. This degradative oxidation can also be induced by reactive oxygen species (ROS), which are generated for example by activated macrophages^38^.

Here, we develop a CPN system based on imidazole units, affording highly fluorescent and biodegradable particles. The resulting polymer chains are fully conjugated along the backbone. The particles decompose upon exposure to ROS such as hydrogen peroxide at concentrations that are relevant for in vivo applications^39^. We show how the particles are degraded by activated macrophages in cell culture. The resulting small molecular degradation products are water soluble. The degradable particles can also be surface functionalized with bio-medical homing devices to allow for targeted delivery of the conjugated polymer imaging probe. The development of bio-degradable CNPs presents a stepping-stone for rational in vivo application of conjugated polymer particles and potential transfer into the clinic.

## Results

### Synthesis of degradable CPNs

**Fig. 1:**
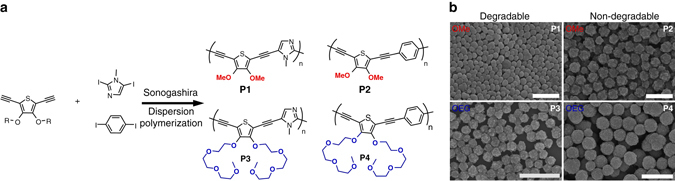
Conjugated polymer nanoparticle synthesis. Sonogashira dispersion polymerization of thiophene monomers with imidazole monomers to produce degradable particles and polymerization with benzene monomers to obtain non-degradable particles. **a** The thiophene monomer carries either methoxy- (OMe, *red*) or oligoethyleneoxy- (OEG, *blue*) side-groups to tune the particle morphology, provide stability for the particles in water and improve the solubility of the degradation products. **b** Scanning electron micrograph of degradable (**P1** and **P3**) and non-degradable (**P2** and **P4**) particles. The *scale bar* represents 1 µm

To incorporate potentially degradable imidazole units into our CPNs, we apply diiodo-*N*-methyl-imidazole. CNPs are produced via Sonogashira dispersion polymerization with an acetylene functionalized thiophene co-monomer to produce uniform particles of the respective fully π-conjugated polymers (see Fig. 1). Alternatively, the functional groups on the monomers can be interchanged. Sonogashira-based step-growth dispersion polymerization has been developed by us and reported previously^12^. In short, particle preparation involves polymerization of the monomers in 1-propanol. This solvent is good for the monomers but poor for the resulting polymers. When a critical molecular weight is reached particle nuclei precipitate from solution and coalesce until they are stabilized by added Triton X-45 and poly(vinylpyrrolidone-*co*-vinylacetate) preventing further particle aggregation. These stabilized nuclei act as seeds for subsequently generated polymer chains reaching the critical molecular weight ( ~ 15 kDa), which condensate onto the particles^40^. This way the particles grow until they reach their final size of a few hundred nanometers in diameter^18^. The size of the particles can be controlled by tuning of the applied monomer concentration. The thiophene monomer unit carries either methoxy- (OMe for **P1**) or oligoethyleneoxy (OEG for **P3**) side-groups to direct the particle morphology, provide stability for the particles in water, and improve the solubility of the resulting degradation products. We apply monomer concentrations to aim for particle sizes at the bottom of the accessible size scale. The variation of the alkoxy periphery entails small differences in the final sizes. We observe particle diameters between 210 and 250 nm (determined via scanning electron microscopy (SEM) image analysis of at least 100 particles). The particles are uniform in size; however, they exhibit rough surfaces as can be seen when imaging the particles in dry state by SEM (see Fig. 1). The rough surface is probably a remnant of the initial nucleation coalescence phase during particle synthesis as well as different tendencies of the rigid backbones and different side groups to crystallize during the condensation phase of the dispersion polymerization^41^. To investigate whether the particles are stable in dispersion, we perform dynamic light scattering (DLS) in water. The CPNs do not aggregate and are individually dispersed. The DLS data corresponds relatively well with the SEM results and gives hydrodynamic diameters of 290 nm for **P1** and 282 nm for **P3** and dispersities of only 5%, respectively (see Supplementary Table 1 and Supplementary Figs. 13 and 15). We also produce particles by copolymerizing *p*-diiodobenzene instead of the degradable imidazole unit to obtain non-degradable particles **P2** and **P4** as controls (see Fig. 1). These particles exhibit hydrodynamic diameters of 492 nm for **P2** and 574 nm for **P4** (Supplementary Table 1 and Supplementary Figs. 14 and 16).

### Degradable imidazole CPNs

**Fig. 2:**
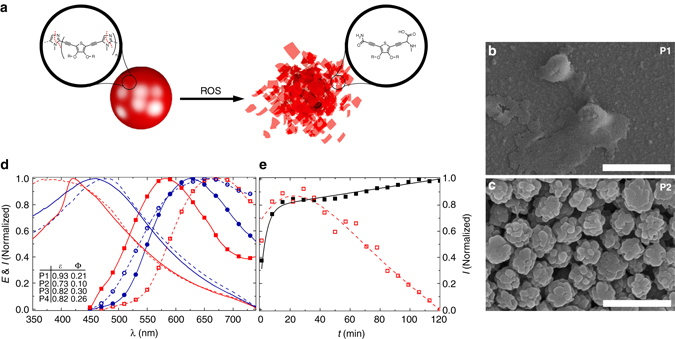
Characterization of the conjugated polymer nanoparticles. **a** Degradative imidazole oxidation of particle by reactive oxygen species to decompose into methylaminoacid and amide terminated 2,5-diethynylene-3,-4-methoxythiophene. **b** Scanning electron micrographs of partially degraded **P1** after treatment with H_2_O_2_ (20 µM in water). The *scale bar* represents 1 µm. **c** Scanning electron micrograph of intact non-degradable **P2** after treatment with H_2_O_2_ (20 µM in water). The *scale bar* represents 1 µm. **d** Extinction and photoluminescence spectra of **P1** (*dashed red*, PL: *open squares*), **P2** (*solid red*, PL: *squares*), **P3** (*dashed blue*, PL *open circles*), and **P4** (*solid blue*, PL: *circles*), all excited at 400 nm. The degradable particles (**P1**, **P3**) show a broad emission tail reaching into the near IR with maxima at around 670 nm. The non-degradable control particles (**P2**, **P4**) are blue shifted with emission maxima at 630 nm (**P2**) and 570 nm (**P4**). The table *inset* gives extinction coefficients *ε* and photoluminescence quantum yields Φ for **P1**–**P4**. **e** Fluorescence intensity of the conjugated particles (at a loading of 0.3 mg/ml) **P1** (*red*) and **P2** (*black*) versus exposure time to H_2_O_2_ (0.3 M). The intensities increase by about 40% caused by a photo-brightening effect. After 20 min, a decrease in the fluorescence intensity of **P1** is apparent, caused by the degradation of **P1**. This decrease in fluorescence intensity is not observed in **P2** particles

To test whether they are degradable, we expose **P1** particles to hydrogen peroxide (H_2_O_2_) at 20 µM in water for 48 h. During this time the turbid dispersion turns transparent. In agreement with the previously described imidazole oxidation^35^, we expect the particles to decompose into methylaminoacidyl- and amide terminated 2,5-diethynylene-3,4-methoxythiophene (see Fig. 2a). When we perform ^1^H-NMR spectroscopy of the degradation products, we indeed find the respective signals for the methine hydrogen, the amines and the acid hydroxyl group proving scission of the imidazole ring (see Fig. 2a and Supplementary Fig. 9). When we expose the **P1** particles to 20 µM of H_2_O_2_ for only a few minutes and deposit and dry them on a surface for SEM analysis we can capture particles, which are only partially degraded (see Fig. 2b). When performing the same experiment with the non-degradable **P2** particles the shape remains intact (*cf*. Figs. 1 and 2c).

The CNPs exhibit fluorescence in the red spectrum. The imidazole particles (**P1**, **P3**) are deep red with emission maxima at around 670 nm and a broad emission tail reaching into the NIR. By contrast, the control particles are blue shifted in their emission, with maximum intensities of 630 nm for **P2** and at 570 nm for **P4** (see Fig. 2d). This shift is due to different intramolecular electronic effects of the phenylene versus the more electron rich imidazole unit. The methoxy- and OEG functionalization of the polymer chains leads to different molecular aggregation behavior. The OEG side-chains in **P3** will induce *J*-aggregates, with a red-shifted fluorescence maximum compared to the methoxy-functionalized P1 particles^42, 43^. We can also follow the degradation of the CPNs by looking at the decay of the fluorescence maximum, while exposing the particles to a solution of H_2_O_2_.

**Fig. 3:**
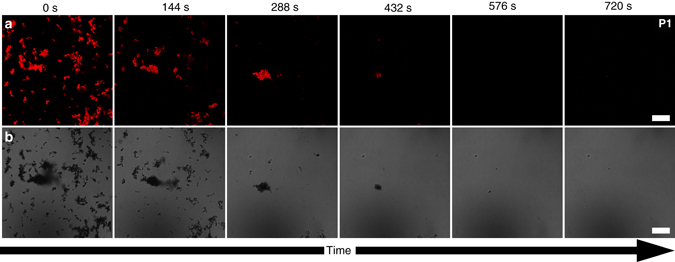
Degradation of the imidazole carrying conjugated polymer nanoparticles. **a** Confocal fluorescence microscopy images and **b** *bright-field* images of degradable particles **P1** exposed to an aqueous 3 M H_2_O_2_ solution. After 12 min the fluorescent particle aggregates disintegrate and individual particles disappear. The *scale bars* represent 20 µm

The degradable particles **P1** are exposed to a 1 wt% (0.3 M) solution of H_2_O_2_ and the fluorescence maximum is recorded over 2 h (see Fig. 2e). We first observe an increase of fluorescence until 20 min into the measurement. Thenceforth, the fluorescence of the particles deteriorates linearly (see red data in Fig. 2e). While the linear decay is expected, the apparent intensity increase by about 40% in the beginning of the measurement is surprising. The increase can either be explained by the fact that through degradation, single chains or lose ends are fully solvated and fluoresce at a higher quantum yield because of reduced intermolecular quenching. Alternatively, the fluorescence intensity increase is caused by a photo-brightening effect, which is induced by the measurement beam and known to occur in conjugated polymers^44^. The latter mechanism seems to be the correct explanation as we also see the photo-brightening effect in the non-degradable **P2** control particles (see *black data* in Fig. 2e). In addition to the fluorescence spectroscopy experiments we also perform confocal fluorescence microscopy to image the CPNs over the course of degradation and rule out unexpected bleaching effects, which could cause the fluorescence decay. The particles exhibit Brownian motion, which is why we image immobilized particles that adhere to the bottom substrate of our imaged specimen (see Fig. 3a and Supplementary Movie 1). Also, here we see the photo-brightening effect, when we plot the fluorescence of the entire field of view versus time (see Supplementary Fig. 12). At the same time we look at the CPNs with bright-field microscopy to also obtain information about aggregates, which are not in the confocal plane (see Fig. 3b and Supplementary Movie 1). It is clearly visible that over the course of only 12 min, the fluorescent particle aggregates disintegrate and the individual particles disappear (see Fig. 3a). Also in the bright-field, the disintegration can be followed until there are no more particles visible. This study proves that the degradation occurs throughout the entire sample and not just at the interface (see Fig. 3b). When we repeat the experiments with non-degradable **P2** particles, the particles remain intact over the entire time of observation. This clearly indicates that the conjugated polymers are cleaved at the imidazole moiety (see Supplementary Movie 2).

### Biodegradation of CPNs

**Fig. 4:**
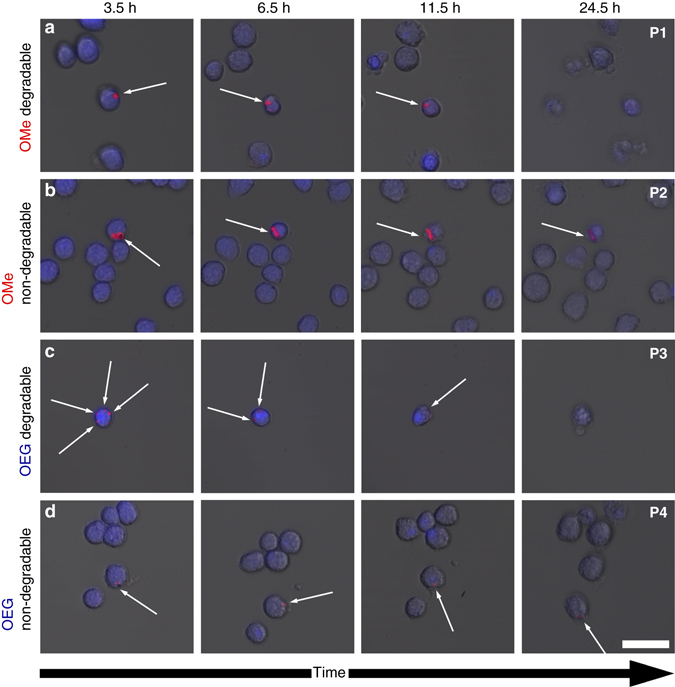
Confocal fluorescence microscopy of macrophages (stained in blue) incubated with degradable and non-degradable particles. The macrophages are exposed to lipopolysaccharides (*LPS*) to activate the macrophages to produce ROS (e.g., H_2_O_2_). The fluorescent particles are highlighted by *white arrows* (Extended time series in Supplementary Fig. 10). **a** The OMe functionalized degradable **P1** particles shrink and the fluorescence signal disappears after 11.5 h. **b** The OMe functionalized non-degradable **P2** particles. **c** The OEG functionalized **P3** particles are degraded and the fluorescence signal disappears after 11.5 h. **d** The OEG functionalized **P4** particles as well as the **P2** particles in **b** do not disintegrate and the fluorescence is visible until the end of the experiment. The faster degradation is caused by the improved solubility in aqueous medium. The *white arrows* indicate where particles are present. The *scale bar* represents 35 µm

**Fig. 5:**
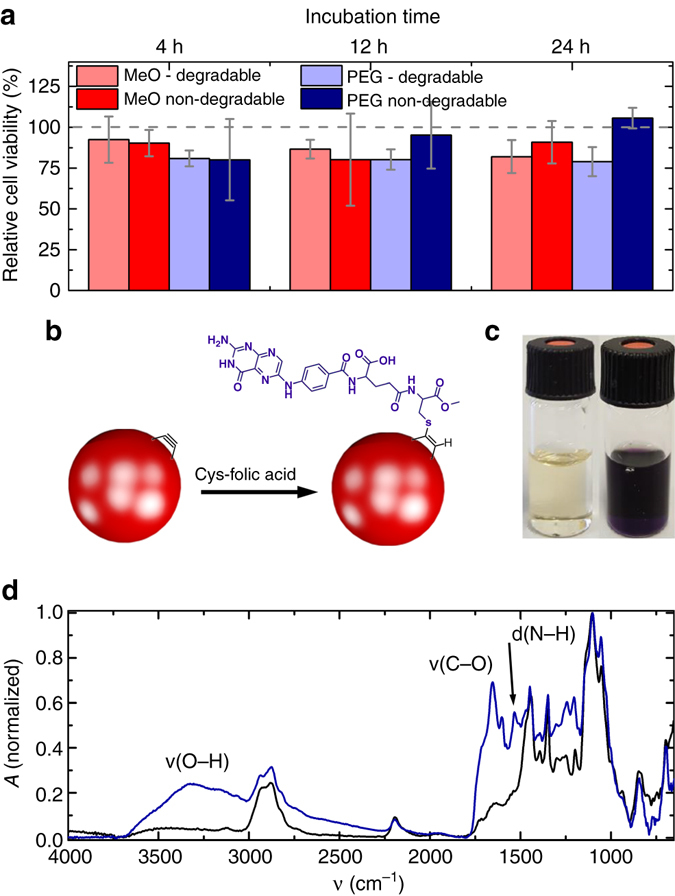
Biocompatibility of the conjugated polymer nanoparticles. **a** Cytotoxicity test: J774A.1 cells are incubated for 4, 12, and 24 h with LPS (particle concentration 5 µg/ml). **b** Acetylene units on the particles are functionalized with cysteine folate using thiol-yne click chemistry. **c** Kaiser test before (*colorless*) and after (*dark-purple*) surface functionalization. The *dark-purple color* illustrates the presence of primary amines. **d** IR spectra before (*black*) and after (*blue*) functionalization. The characteristic band of the amide oscillation is highlighted by an *arrow*

Having proven that fully conjugated polymer particles with imidazole units in the backbone can be degraded using H_2_O_2_ at concentrations beyond biologically relevant levels; we now want to look at bio-degradability of our particles. To investigate biodegradability we add our particles to J774A.1 macrophages in cell culture. The macrophages are exposed to lipopolysaccharides (LPS), which is a well-known stimulant to activate the macrophages to produce ROS, such as H_2_O_2_. The macrophages are stained using a CellTracker™ Blue CMHC dye for better visualization and incubated with our particles for two hours before transferal to the confocal microscope, where we image and follow individual cells with few ingested particles over a period of 3 days (see Fig. 4 and Supplementary Fig. 10). On the confocal microscope the cells are cultivated in FluoroBrite™ medium, which is low in autofluorescence, in a CO_2_ rich atmosphere of 5% and at a temperature of 37.5 °C. For all samples we observe that the macrophages appear healthy for the first 12 h with some macrophages starting to have disrupted cell membranes at a time of 24 h. At the applied concentration (4 µg/ml) the degradation products do not seem to be toxic, at least not over the course of the experiment. To validate these hypotheses, we perform a cytotoxicity study under more controlled cell culture conditions (i.e., in a dedicated incubator, with controlled humidity and CO_2_ level). We compare the FluoroBrite™ medium to standard DMEM medium and test also for higher particle concentrations of up to 100 µg/ml (see Supplementary Fig. 11). We determine relative cell viabilities with respect to LPS activated macrophages incubated in FluoroBrite™, which have not been exposed to degradable particles (as a control). We observe good relative cell viabilities between 80 and 100% for macrophages exposed to 5 µg/ml of particles over a period of up to 24 h, independent of their alkoxy-functionalization or their degradability (see Fig. 5a and Supplementary Fig. 11b–d). This concentration is similar to the one used during the confocal microscopy study (4 µg/ml). At high particle concentrations of 100 µg/ml the viability of macrophages with ingested degradable particles is lower than for the non-degradable particles. This suggests some toxicity of the degradation products at high concentrations. All macrophages enter apoptosis after about 24 h into the experiment. This effect is not due to the particles but can be explained by the high concentrations of ROS, which rises in LPS-activated macrophages and eventually triggers apoptosis^45^.

We see that during the confocal microscopy study OMe functionalized **P1** particles shrink and the fluorescence signal disappears after  ~ 11.5 h (see Fig. 4a). Similarly, the **P3** particles are degraded; however, here some of the particles disintegrate even at earlier points in time (see Fig. 4c). We account this faster degradation to the improved solubility of **P3**-fragments in aqueous media mediated through the OEG functionalization. By contrast, the non-degradable particles do not disintegrate and no reduction in size is observable (see Fig. 4b, c and Supplementary Fig. 10). This proves that our imidazole functionalized CPNs are degradable in macrophages at biologically relevant levels of ROS.

Such particles could now in principle be used for imaging of malignant tumors, which are rich in tumor-associated macrophages (TAMs)^46^. The particles can accumulate in the tumor tissue through the enhanced permeability and retention effect. Here, they could then be taken up and degraded by the TAMs. For specific targeting of TAMs by the particles, the folic acid motif could be used to target the folate receptor β, which is expressed on TAMs^47^. Here, we show that we can functionalize the surface of our degradable CPNs with cysteine-terminated folic acid recognition motifs using thiol-yne click-chemistry (see Fig. 5b)^12^. We continue with the OEG functionalized **P3** particles and perform the attachment of cysteine functionalized folic acid to the surface of the particles in a mixture of degassed dimethyl sulfoxide and water. The folic acid compound is added in large molar excess and the mixture is stirred while irradiating with 365 nm ultraviolet (UV) light from an 8 W hand lamp to allow thiol-yne click coupling. The particles are then purified by centrifugation, exchange of the supernatant, and redispersion. To test the successful attachment of the folate motif to the surface of the particles, we perform the Kaiser-test to probe for primary amines, which are part of the folic acid compound. We purify the particles until the Kaiser-test is negative for the supernatant. Then we add the Kaiser-reagent to the aqueous dispersions of our **P3** particles, which turns dark purple, proving the existence of free amines on the particle surface (see inset Fig. 5b). To confirm our assumption, that this observation is due to the surface-attached folic acid, we perform IR spectroscopy on our particles before and after folic acid functionalization. We find characteristic vibrational bands for hydroxyl (3324 cm^−1^), amide carbonyl (1654 cm^-1^), and amide N-H (1535 cm^−1^) groups, which are absent in the non-functionalized particles (see Fig. 5b). Furthermore, we determine the ζ-potential of the particles before (−3.84 mV) and after functionalization with the folic acid moiety (−8.71 mV). We perform the measurements at high pH to deprotonate the carboxylic acid moiety. Using the formula derived by Oshima et al.^48^ for the surface charge density of sub-micron polymer particles and assuming that each folic acid molecule contributes one negative charge, we obtain a particle coverage of  ~ 1000 folic acid molecules per particle (1 molecule per 250 nm^2^). These results substantiate our claim for surface functionalization.

## Discussion

In conclusion, we have developed a concept to provide fully conjugated polymer particles with bio-degradability, through integration of imidazole units. The ROS concentration in stimulated macrophages is sufficient to degrade the particles. The particles can be surface functionalized for labeling of specific biological recognition motifs for targeted imaging. In the future, it needs to be confirmed that the ROS concentration for degradation of the particles is sufficient in vivo. The concept of oxidative degradation could be extended by loading the hydrophobic conjugated polymer particles with drugs, which can be released upon degradation of the particles by macrophages at a specific site. In contrast to fluorescent degradable inorganic materials, the conjugated polymer particles provide more optical gain because of the dense luminophore packing and, therefore, have improved signal-to-noise ratios^49, 50^. The approach opens a wide variety of applications for conjugated polymer particles in the biological and medical fields such as imaging, drug-delivery, and theranostics.

## Methods

### Dynamic light scattering

The DLS measurements are performed at 25 °C on a Zetasizer Nano ZS from Malvern Instruments. The samples are prepared by diluting 100 μl of particle dispersions with 5 ml of deionized water. The cumulants analysis of the autocorrelation function obtained by the DLS measurements is defined by the International Standard on Dynamic Light Scattering ISO13321 and ISO22412. This analysis provides a mean value for the size (z-average) and a width parameter for the polydispersity index. Every determined diameter and dispersity value has been averaged over three measurements.

### Gel permeation—size exclusion chromatography (SEC)

Molecular weights and molecular weight distributions were determined by SEC. SEC analyses were carried out with tetrahydrofuran as eluent using a HPLC pump (PU-2080plus, Jasco) equipped with a refractive index detector (RI-2031plus, Jasco) and an evaporative light scattering detector (PL-ELS-1000, Polymer Laboratories). The sample solvent contained 250 mg/ml 3,5-di-tert-4-butylhydroxytoluene (≥ 99%, Fluka) as a stabilizer for THF and as an internal standard. One pre-column (8 × 50 mm) and four SDplus gel columns (8 × 300 mm, MZ Analysentechnik) were applied at a flow rate of 1.0 ml/min at 20 °C. The diameter of the gel particles measured 5 μm, the nominal pore widths were 50, 10^2^, 10^3^_,_ and 10^4^ Å. Calibration was achieved using polystyrene standards (Polymer Standards Service).

### Mass spectrometry

Mass spectra are acquired on a Finnigan SSQ7000 (EI, 70 eV) spectrometer and on a ThermoFinnigan LCQ Deca XP plus (ESI) spectrometer.

### Kaiser test

The Kaiser test is performed by preparing the following three agents: (a) aqueous KCN solution (1.0 ml, *β* = 0.66 mg/ml) in pyridine (49.0 ml) (b) ninhydrin (1.0 mg, 5.61 mmol) in *n*-butanol (20.0 ml) (c) phenol (20.0 g, 0.213 mol) in *n*-butanol (10.0 ml) A few droplets of each agent are added to an alkaline sample and the mixture is heated to 80 °C for a few minutes. The presence of primary amino groups is indicated by a dark blue to purple color. (In case of a negative test, the mixture remains yellow).

### Confocal microscopy

Confocal laser scanning microscopy is performed on a Leica TCS SP8 to visualize the time-resolved degradation of the conjugated particles as well as the in-vitro cell experiments using prepared macrophages. The conjugated particles are excited with the wavelength of *λ* = 561 nm. The sample chamber is the well of a 96-well microplate for the time-resolved degradation with H_2_O_2_ (3 M). For the time-resolved degradation by macrophages, the sample chamber is the well of a Cellstar cell culture flask (Greiner), which is then placed in a TOKAI HIT stage top incubator (INUBG2E-GSI) so that a stable 5% CO_2_ + 95% air mixed gas supply as well as a temperature of 37.5 °C can be adjusted. For long-term experiments the well is temporary sealed with parafilm.

### Microscopic macrophage degradation study

J774A.1 (ATCC) cells are seeded in Cellstar cell culture flasks (Greiner) and cultured in FluoroBrite (Biochrom) + 10% FBS (Invitrogen) + 1% penicillin/streptomycin (Invitrogen). Stimulation of cells is achieved by adding LPS (Sigma Aldrich) 1 µg/ml. The cells are incubated with the degradable and non-degradable particle dispersions (4 µg/ml) for two hours and subsequently the dispersions are exchanged with cell culture medium. The cells are stained with CellTracker^TM^ Blue CHMC Dye (Thermo Fisher).

### Cytotoxicity

The toxicity of the particles and their degradation products on J774A.1 is investigated by trypan blue staining. In all, 3 × 10^4^ cells are seeded in 24-well plates (Corning) and cultured in Fluorobrite (Biochrom) + 10% FBS (Invitrogen) + 1% penicillin/streptomycin (Invitrogen) and stimulated by adding 1 µg/ml LPS (Sigma Aldrich) for 1 h. Cells incubated with cell culture medium without LPS served as negative controls. Degradable and non-degradable particles are diluted in cell culture medium and added to the cells with a concentration of either 5 or 100 µg/ml and the cells are incubated for 4, 12, 24, and 48 h. Three samples per condition are analyzed. After incubation, the cells are removed with a cell scraper (Falcon) and transferred to eppendorf tubes. The cell suspension is centrifuged and the supernatant is removed. The cell pellet was dissolved in 50 µl cell culture medium. Twenty-five microliters of the cell suspension is mixed with 25 µl Trypan blue staining solution (Gibco). Trypan blue positive cells (blue cells) were counted using Cedex XS cell counter (Innovates AG). The percentage of positive cells as a function of the total cell number was calculated.

### Photoluminescence spectroscopy and quantum yield (Φ)

Photoluminescence spectroscopy measurements are conducted on a Horiba Jobin-Yvon FluoroMax-4 spectrofluorometer with a 150 W xenon lamp, ozone free. The software has been FluorEssenceTM version 3.8.2.2, Origin version 8.6001.The conjugated particles are dispersed in water and in case of the degradation studies H_2_O_2_ is added, leading to a H_2_O_2_-concentration of 1.1 wt%.

### UV–Visible spectroscopy

Extinctions are measured with a Varian Cary 50 Bio UV–Visible spectrophotometer. The software is Varian UV Cary Scan 3.00(303).

### Scanning electron microscopy

Field emission scanning electron microscopy is performed on a S-4800 microscope (Hitachi) to determine the size and shape of the synthesized conjugated particles. Before imaging, particle dispersions are deposited and dried on silicon wafer (CrysTec GmbH). All samples are sputtered with a thin layer of Au/Pd.

### Data availability

All data are available from the corresponding authors upon reasonable request.

## Electronic supplementary material

Supplementary Information

Supplementary Movie 1

Supplementary Movie 2
