# Supplementary material for: Bio-degradable highly fluorescent conjugated polymer nanoparticles for bio-medical imaging applications
Source: Nat Commun. 2017 Sep 7;8:470. doi: 10.1038/s41467-017-00545-0 (PMC5589938; doi:10.1038/s41467-017-00545-0)
Supplement: Supplementary file 1 — Supplementary Information [file 41467_2017_545_MOESM1_ESM.pdf]

Description of Supplementary Files

File Name: Supplementary Information
Description: Supplementary Figures, Supplementary Table and Supplementary Methods

File Name: Supplementary Movie 1
Description: Degradation study of P1 particles in 3 M of aqueous H2O2. Brightfield (left) and confocal fluorescence channel (right) are recorded over a time of 12 min. The degradation and solvation of the conjugated polymer particles is clearly apparent.

File Name: Supplementary Movie 1
Description: Stability study of P2 particles in 3 M of aqueous H2O2. Brightfield (left) and confocal fluorescence channel (right) are recorded over a time of 60 min. The pulsation in the fluorescence channel is an artefact from the autofocus function, which is enabled to remain in focal plane with maximum fluorescence. The results indicate that the particles are not susceptible to H2O and remain stable over the course of the experiment.

Supplementary Methods:

2,5-Dibromo-3,4-dimethoxythiophene (1)
3,4-dimethoxythiophene (6.05 g, 41.9 mmol, 5.0 mL) is dissolved in chloroform (75 mL) and acetic acid (75 mL) and subsequently cooled to 0 °C and degassed by bubbling with argon. N-Bromosuccinimide (18.66 g, 104.8 mmol, 2.5 eq) is added slowly and the slightly yellow solution is stirred at 0 °C for another three hours to the exclusion of light. Subsequently, the reaction solution is stirred at room temperature over night.
The resulting dark brown solution is quenched with water (50 mL) and the aqueous phase is extracted with DCM (3 x 100 mL). The united organic phase is dried with Na_2SO_4 and purified via column chromatography ($CHCl_3$:Petroleum ether 3:2). After removing the solvent under reduced pressure, the product is obtained as a brown liquid. Yield: 9.2423 g (30.61 mmol, 73.0 %).

^{1}H-NMR ($CDCl_3$, 400 MHz, 25 °C): δ 3.91(s, 6H,O-C*H*$_3$) ppm. ^{13}C[^{1}H]-NMR (CDCl3, 101 MHz): δ 148.10 (s), 94.95 (s), 61.09 (s), 61.01 (s) ppm. MS (ESI): m/z (%) = 340.8 (100, $[MK]^+$, $C_6H_6Br_2O_2S+K^+$).

3,4-Dimethoxy-2,5-di[(trimethylsilyl)ethynyl]thiophene (2)
(**1**) (8.9203 g, 27.45 mmol) is dissolved in THF (250 mL) and degassed by bubbling with argon for 15 min. Subsequently the solution was mixed with CuI (130.7 mg, 0.6863 mmol, 0.0025 eq) and $Pd(PPh_3)Cl_2$ (483.5 mg, 0.6863 mmol, 0.025 eq) while keeping an argon atmosphere. Dry diisopropylamine (20 mL) is added and afterwards trimethylsilylacetylene (8.0892 g, 82.36 mmol, 11.7 mL, 3 eq). The solution is stirred for five days to the exclusion of light. The completion of the reaction is controlled by TLC.
The dark brown suspension is washed with a saturated NH_4Cl-solution (2 x 30 mL) and with water (2 x 30 mL). The aqueous phase is extracted with DCM (3 x 50 mL) and the united organic phase is dried over Na_2SO_4. The raw product is purified by column chromatography (pentane: DCM / 4:1). After removing the solvent, the product is obtained as an orange-yellow solid. Yield: 7.2245 g (21.50 mmol, 78.3 %)

^{1}H-NMR (CDCl3, 400 MHz, 25 °C): δ 4.13 (s, 6H, O-CH_3), 0.22 (s, 18H, Si-(C*H*$_3$)$_3$) ppm. ^{13}C[^{1}H]-NMR (CDCl3, 101 MHz, 25 °C): δ 150.12 (s), 102.16 (s), 102.07 (s,), 96.00 (s,), 59.92 (s,), - 0.18 (s,) ppm. MS (ESI): m/z (%) = 337.1 (100, $[M]^+$, $C_{16}H_{25}O_2SSi_2^+$).

2,5-Diethynyl-3,4-dimethoxythiophene (M1)
(**2**) (182.4 mg, 0.5427 mmol) is dissolved in methanol (60 mL) and DCM (30 mL). The solution is degassed by bubbling with argon for 15 min. Subsequently, K_2CO_3 (315.0 mg, 4.2 eq) is added to the reaction mixture and stirred at room temperature over night to the exclusion of light.
The slightly yellow solution is washed with water (50 mL) and brine (50 mL). The aqueous phase is extracted with DCM (3 x 25 mL) and the combined organic phase is dried over $MgSO_4$. After removing the solvent under pressure the desired product is obtained as a brownish liquid. Yield: 0.1017 g (0.5296 mmol, 97.6 %).

^{1}H-NMR (CDCl3, 400 MHz, 25 °C): δ 4.13 (s, 6H, O-C*H*$_3$), 3.40 (s, 2H, C*H*) ppm. ^{13}C[^{1}H]-NMR (CDCl3, 101 MHz, 25 °C): δ 150.63 (s), 101.28 (s), 84.19 (s), 75.31 (s), 60.11 (s) ppm. MS (ESI): m/z (%) = 199.2 (100, $[MLi]^+$, $C_{10}H_8O_2S+Li^+$).

3,4-Di[2-[2-(2-methoxyethoxy)ethoxy]ethoxy]thiophene (3)
3,4-dimethoxythiophene (1.814 g, 12.58 mmol, 1.5 mL) is dissolved in triethylene glycol monomethyl ether (5.507 g, 33.54 mmol, 5.4 mL, 2.7 eq) and degassed by bubbling with argon (15 min). While keeping an argon atmosphere *p*-toluene sulfonic acid is added to the reaction mixture. Afterwards, the solution is stirred for three days at 90 °C under constant nitrogen flow. The raw product is purified using column chromatography (ethyl acetat: dimethoxyethane / 9.5:0.5). After removing the solvent *in vacuo*, the product is obtained as a yellow liquid. Yield: 2.857 g (6.999 mmol, 55.7 %).

^{1}H-NMR (CDCl3, 400 MHz, 25 °C): δ 6.20 (s, 2H, C*H*), 4.11 (m, 4H, C*H*$_2$), 3.81 (m, 4H, C*H*$_2$), 3.70 (m, 4H, C*H*$_2$), 3.64 (m, 8H, C*H*$_2$), 3.51 (m, 4H, C*H*_$_2$), 3.34 (s, 6H, O-C*H*_$_3$) ppm. ^{13}C[^{1}H]-NMR (CDCl3, 101 MHz): δ 147.26 (s), 98.05 (s), 72.08 (s), 70.80 (s), 70.70 (s), 69.98 (s), 69.65 (s), 59.19 (s) ppm.

2,5-Diiododo-3,4-di[2-[2-(2-methoxyethoxy)ethoxy]ethoxy]thiophene (M3)
(**3**) (675.4 mg, 1.655 mmol) and p- toluene sulfonic acid (30.7 mg, 0.1614 mmol, 0.1 eq) are dissolved in ethanol (5 mL9) and degassed by bubbling with argon for 10 min. Subsequently, N-iodosuccinimde is added under continuous argon flow and stirred for three days to the exclusion of light and air.
The reaction mixture is quenched with sat. $Na_2S_2O_3$-solution (6 mL), ethyl acetat (6 mL) and water (6 mL). The solvent of the organic phase is removed under reduced pressure and the raw product is dissolved in DCM (25 mL). Afterwards the solution is washed with water (25 mL) and brine (25 mL) and dried over $MgSO_4$. After removing the solvent *in vacuo* the pure product is obtained as a dark brown oil. Yield: 0.8203 g (1.242 mmol, 75.1 %).

^{1}H-NMR (DMSO-*d*6, 400 MHz, 25 °C): δ 4.11 (m, 4H, C*H*$_2$), 3.67 (m, 4H, C*H*$_2$), 3.56 (m, 4H, C*H*$_2$), 3.52 (m, 8H, C*H*$_2$), 3.43 (m, 4H, C*H*$_2$), 3.24 (s, 6H, O-C*H*$_3$) ppm. ^{13}C[^{1}H]-NMR (CDCl3, 101 MHz): δ 177.40 (s), 151.27 (s), 72.75 (s), 72.05 (s) 70.80 (d), 70.68 (s), 70.15 (s), 59.14 (s), 29.69 (s) ppm. MS (ESI): m/z (%) = 661 (100, $[M]^+$, $C_{19}H_{31}I_2O_8S^+$).

.

2,5-Di[(trimethylsilyl)ethynyl]-1*H*-methyl-imidazole (4)

2,5-diiodo-1-methyl-1*H*-imidazole (568.3 mg, 1.668 mmol) is dissolved in THF (90 mL) and degassed by bubbling with argon for 15 min. Subsequently the solution is mixed with CuI (33.0 mg, 0.1733 mmol, 0.1 eq) and $Pd(PPh_3)Cl_2$ (118.7 mg, 0.1733 mmol, 0.1 eq) while keeping an argon atmosphere. Dry diisopropylamine (30 mL) is added and afterwards trimethylsilylacetylene (360.4 mg, 3.670 mmol, 519 µL, 2.2 eq). The solution is stirred at 50 °C over night. The completion of the reaction is controlled by TLC.

Subsequently, the solvent is removed under reduced pressure and the raw product is purified by column chromatography (ethyl acetate: DCM / 1:5). After removing the solvent *in vacuo*, the product is obtained as a pure brownish powder. Yield: 0.2729 g (0.9941 mmol, 59.6 %).

^{1}H-NMR (CDCl3, 300 MHz, 25 °C): δ 7.22 (s, 1H, C*H*), 3.65 (s, 3H, N-C*H*3), 0.25 (d, 18H, Si-(CH_3)$_3$) ppm. ^{13}C[^{1}H]-NMR (CDCl3, 101 MHz): δ 117.57 (s), 32.52 (s), 29.88 (s), 0.19 (s), 0.01 (s), 0.05 (s) ppm. MS (ESI): m/z (%) = 275.1 (100, $[M]^+$, $C_{14}H_{23}N_2Si_2^+$).

2,5-Diethynyl-1*H*-methyl-imidazol (M4)

(**5**) (69.4 mg, 0.2062 mmol) is dissolved in methanol (80 mL) and DCM (40 mL). The solution is degassed by bubbling with argon for 15 min. Subsequently, K_2CO_3 (125.4 mg, 0.907 mmol, 4.4 eq) is added to the reaction mixture and stirred at room temperature over night to the exclusion of light and air.

The reaction mixture is washed with water (50 mL) and brine (50 mL). The aqueous phase is extracted with DCM (3 x 25 mL) and the combined organic phase is dried over $MgSO_4$. After removing the solvent under pressure the desired product was obtained as a brownish liquid. Yield: 32.2 mg (0.2474 mmol, 97.9 %).

^{1}H-NMR (CDCl3, 400 MHz, 25 °C): δ 7.25 (s, 1H, C*H*), 3.65 (s, 3H, N-CH_3), 3.48 (s, 1H, C*H*), 3.33 (s, 1H, C*H*) ppm. ^{13}C[^{1}H]-NMR (CDCl3, 101 MHz): δ 134.82 (s), 85.16 (s), 81.84 (s), 72.68 (s), 71.59 (s), 32.06 (s), 29.42(s) ppm. MS (ESI): m/z (%) = 131.06 (100, $[M]^+$, $C_8H_7N_2^+$).

SONOGASHIRA dispersion polymerization

The two bifunctional monomers (0.080 mmol) are dissolved in *n*-propanol (6.6 mL) with the aid of ultrasonication. The solution is mixed with PVPVA (720 mg) and Triton® X-45 (800 mg) and stirred at 50 °C until the stabilizers are dissolved. Subsequently, the solution is filtrated using a microporous filter (CHROMAFIL® XTRA PTFE-42/25) and degassed by continuous argon flow. While keeping an argon atmosphere, the catalyst $Pd(PPh_3)_2Cl_2$ (1.5 mg, 2.1 µmol, 0.03 eq.) and co-catalyst CuI (3.0 mg, 16 µmol, 0.2 eq.) are added and the resulting dispersion is stirred at 70 °C. As soon as all reactants are dissolved, the reaction is started by addition of degassed aqueous triethylamine (*wt*% = 15 %, 1.6 mL) and stirred at 70 °C for three hours. Afterwards, the dispersion is cooled to room temperature and centrifuged. The continuous phase is decanted and the residue is redispersed in *n*-propanol through ultrasonication. This procedure is repeated six times.

P1: ^{1}H-NMR (CDCl3, 400 MHz, 25 °C): δ 7.28 (s, 1H, C*H*), 5.34 (br s, 3H, CH_3), 4.29 (br s, 6H, CH_3) ppm.

P2: ^{1}H-NMR (CDCl3, 400 MHz, 25 °C): δ 7.59-7.43 (m, 4H_{aryl}, C*H*), 4.22 (br s, 6H, CH_3) ppm.

P3: ^{1}H-NMR (CDCl3, 400 MHz, 25 °C): δ 7.29 (s, 1H, C*H*), 5.46 (br s, 3H, CH_3), 4.48-3.69 (m, 18H, CH_3+CH_2) ppm.

P4: ^{1}H-NMR (CDCl3, 400 MHz, 25 °C): δ 7.98-7.42 (m, 2H_{aryl}, C*H*), 3.96-3.39 (m, 15H, CH_3+CH_2) ppm.

Cysteine folate

Folic acid (31.9 mg, 0.0723 mmol) is dissolved in DMSO (10.0 mL) and mixed with triethylamine (3.7 mg, 0.0365 mmol, 5.09 µL, 0.5 eq.). Afterwards, Boc_2O (0.0863 mmol, 18.8 mg, 19.83 µL, 1.2 eq.) is added and the resulting solution is stirred overnight to the exclusion of light. The yellow solution is mixed with water (1.0 mL) and extracted using diethyl ether (3 x 10.0 mL). Afterwards, EDC·HCl (13.9 mg, 0.0723 mmol, 1 eq.) and NHS (8.3 mg, 0.0723 mmol, 1 eq.) are added and the solution is stirred at room temperature (20 minutes). Subsequently, the solution is mixed with cysteine monomethyl ether (12.4 mg, 0.0723 mmol, 1 eq.) and stirred for two days to the exclusion of light. Afterwards, the solution is mixed with aqueous HCl solution (1.0 mol·L-1, 100 µL) and stirred for one more day. The product is used in solution without further purification.

Surface functionalization with cysteine folate

A glass vial with rubber septum is charged with a mixture of conjugated polymer particles (2.0 mg) in n-propanol (500 µL), 2,2-dimethoxy-2-phenylacetophenone (30.0 mg) and distilled water (2.5 mL). The reaction mixture is degassed for at least ten minutes by bubbling with argon. Cysteine folate (6, 3.2 mg) in DMSO (2.0 mL) is injected to the reaction mixture and degassed again for five minutes. The reaction mixture is irradiated using a UV lamp with a wavelength of λ = 365 nm at room temperature under magnetic stirring. The particles are purified by washing with DMSO and n-propanol several times until the Kaiser test of decanted supernatant indicates the absence of free amines.

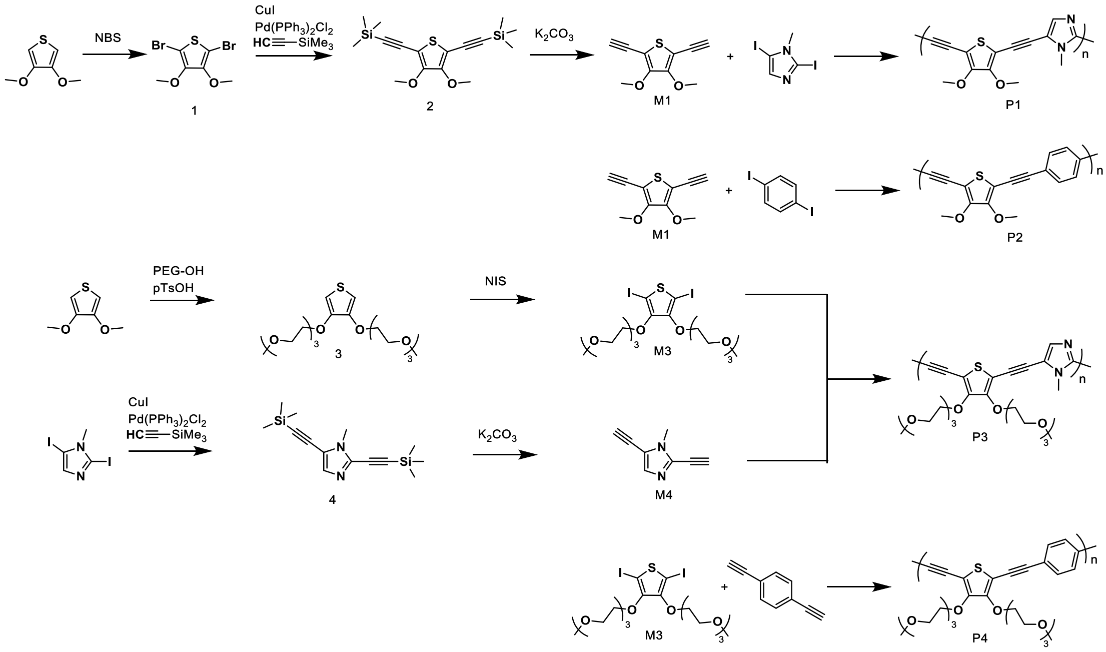

Supplementary Figure 1: Reaction equations for the synthesis of the monomers and polymer particles.

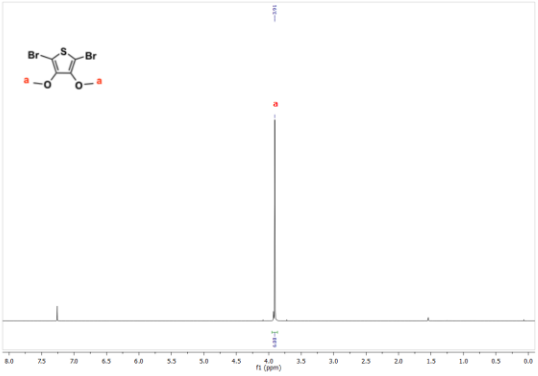

Supplementary Figure 2: ^{1}H-NMR of **1**.

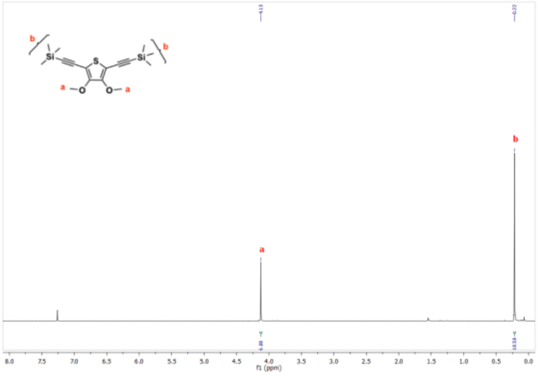

Supplementary Figure 3: ^{1}H-NMR of **2**.

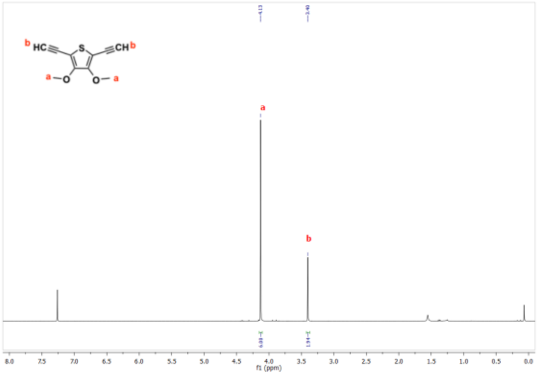

Supplementary Figure 4: ^{1}H-NMR of **M1**.

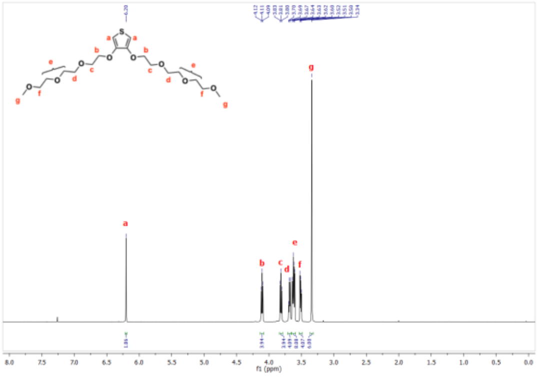

Supplementary Figure 5: ^{1}H-NMR of **3**.

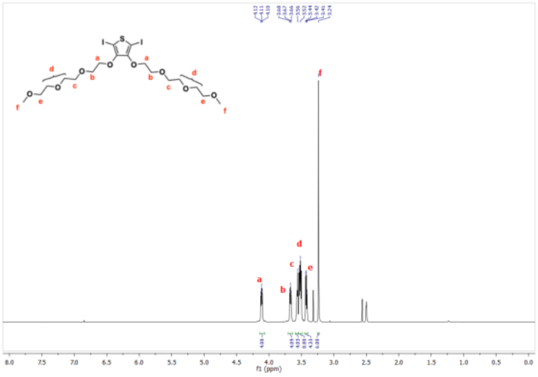

Supplementary Figure 6: ^{1}H-NMR of **M3**.

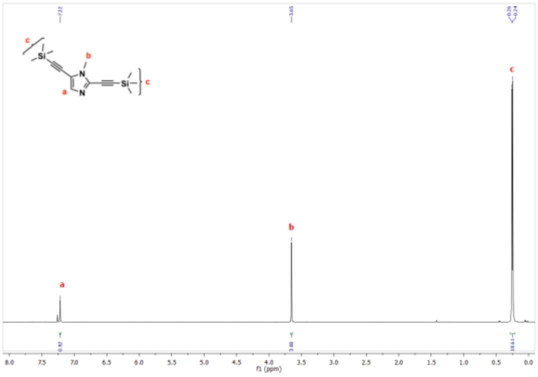

Supplementary Figure 7: ^{1}H-NMR of **4**.

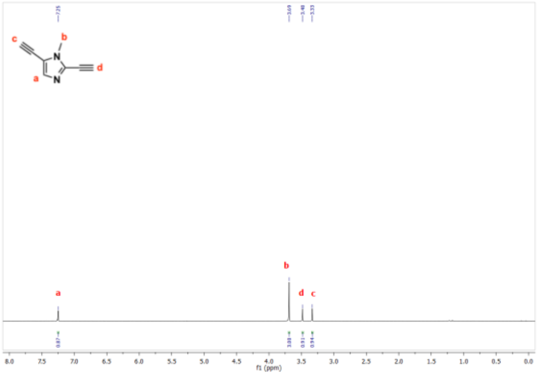

Supplementary Figure 8: ^{1}H-NMR of **M4**.

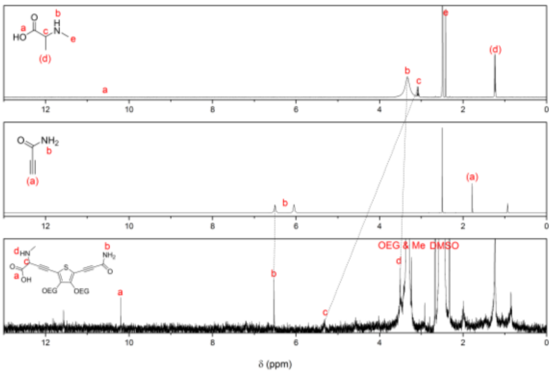

Supplementary Figure 9: ^{1}H-NMR in DMSO-d6 of the crude degraded **P3** particles and fragments for comparison.

Supplementary Table 1: Properties of the conjugated polymer particles. Diameter d, standard deviation σ, hydrodynamic diameter d_{Rh}, extinction coefficient ε and photoluminescence quantum yield Φ.

type	d_{SEM} (nm)	σ_{SEM} (nm)	d_{Rh} (nm)	ε	Φ (%)	M_w (kDa)	*PDI*
**P1**	209.5	±22.7	290.4±0.05	0.93 ± 0.05	21.05	2.02	1.1
**P2**	435.9	±31.0	491.6±0.03	0.72 ± 0.05	10.23	4.75	2.2
**P3**	248.0	±22.8	283.4±0.05	0.82 ± 0.02	29.87	2.14	1.1
**P4**	487.5	±45.0	573.6±0.10	0.82 ± 0.01	25.63	3.57	1.2

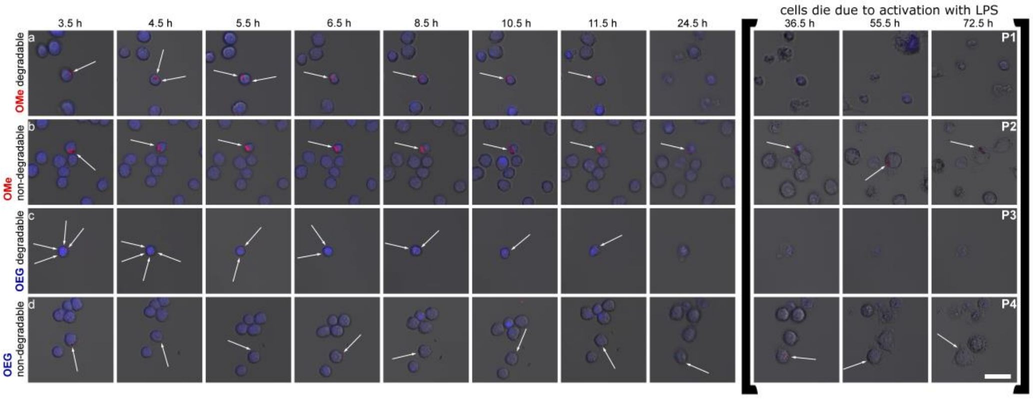

Supplementary Figure 10: Confocal microscopy degradation study: The macrophages are exposed to lipopolysaccharides (LPS) to activate the macrophages to produce ROS (e.g. H_2O_2). The scale bar represents 35 µm.

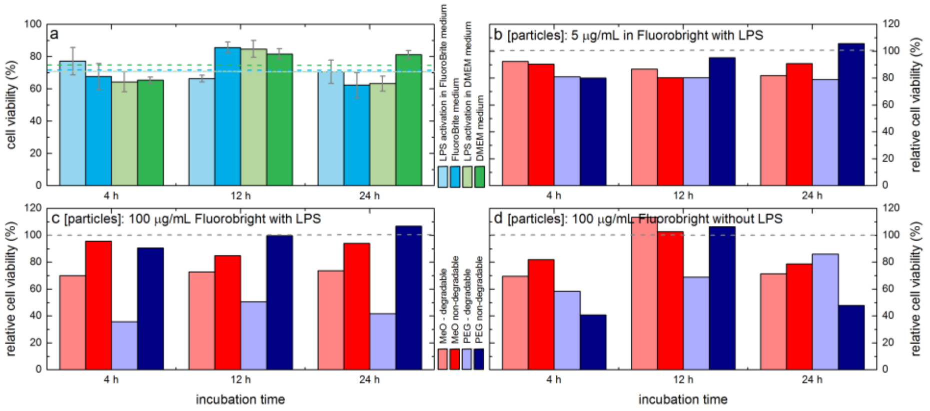

Supplementary Figure 11: Cytotoxicity tests: a) Cell viability of activated and non-activated macrophages at three points in time (4, 12, 24 h) in different cell media: light blue: LPS activation in FluoroBrite medium, blue: non-activated in FluoroBrite medium, light green: LPS activated in DMEM medium and green: non-activated in DMEM medium. The dotted lines represent the overall average values (between 71 and 76 %). b-d) represent relative cell viabilities, where macrophages incubated with particles are compared to macrophages without particles to rule out the influence of FluoroBrite medium: light red: degradable particles with methoxy-thiophene, red: non-degradable methoxy-thiophene particles, light blue: degradable particles based on OEGylated thiophenes and in blue: non-degradable particles with OEGylated thiopnene. (b) Shows the relative cell viabilities for LPS activated macrophages incubated with 5 µg/mL particles, (c) shows the relative cell viabilities of LPS activated macrophages for a particle concentration of 100 µg/mL and (d) shows the relative cell viabilities for non-activated macrophages for 100 µg/mL of particles.

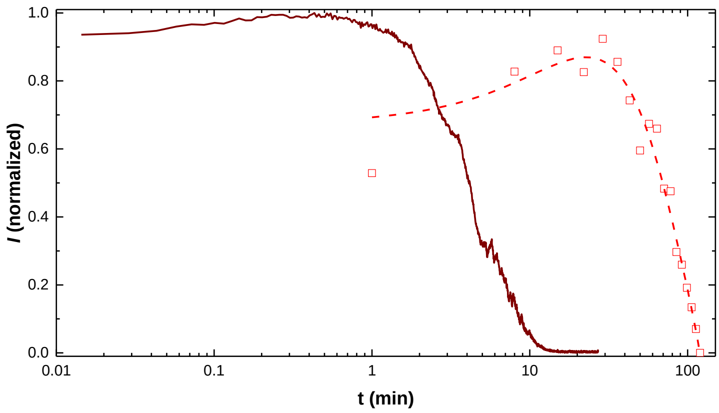

Supplementary Figure 12: Normalized fluorescence intensity at λ_{max} of **P1** treated with H_2O_2 over time in the confocal microscopy (dark red, 3 M H_2O_2) and during photoluminescence spectroscopy (red squares and dashed line, 0.3 M H_2O_2).

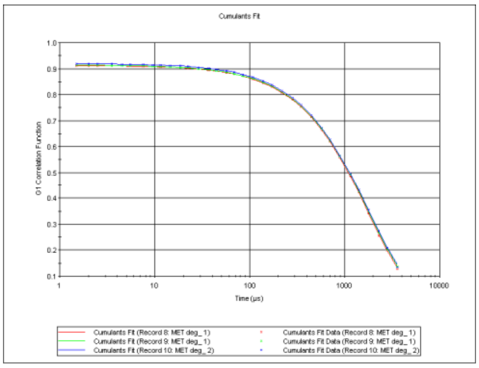

Supplementary Figure 13: Representative correlation functions for the DLS measurements of **P1**.

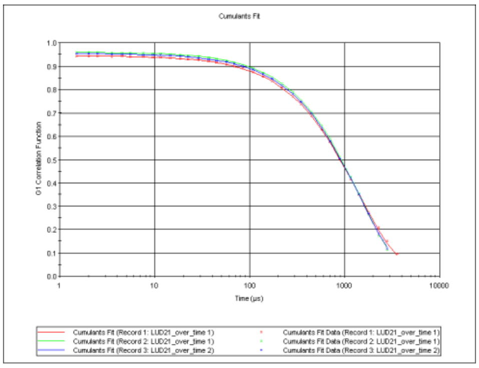

Supplementary Figure 14: DLS measurements of **P2**.

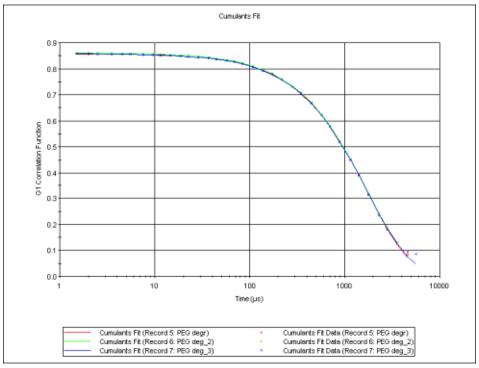

Supplementary Figure 15: DLS measurements of **P3.**

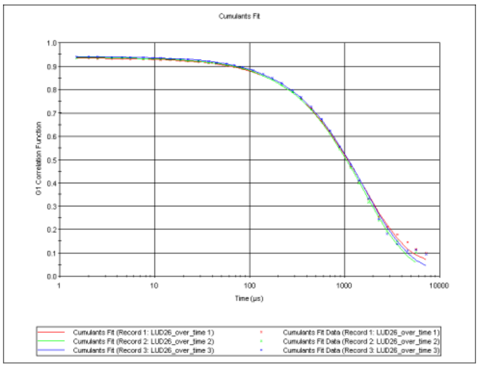

Supplementary Figure 16: DLS measurements of **P4**.
